# TOXiTAXi: a web resource for toxicity of *Bacillus thuringiensis* protein compositions towards species of various taxonomic groups

**DOI:** 10.1038/s41598-020-75932-7

**Published:** 2020-11-13

**Authors:** Jakub Baranek, Bartłomiej Pogodziński, Norbert Szipluk, Andrzej Zielezinski

**Affiliations:** 1 Department of Microbiology, Faculty of Biology, Adam Mickiewicz University in Poznan, Uniwersytetu Poznanskiego 6, 61-614 Poznan, Poland; 2 Department of Computational Biology, Faculty of Biology, Adam Mickiewicz University in Poznan, Uniwersytetu Poznanskiego 6, 61-614 Poznan, Poland

**Keywords:** Applied microbiology, Bacteria, Pathogens, Entomology, Databases, Software, Plant biotechnology

## Abstract

Bioinsecticides consisting of different sets of *Bacillus thuringiensis* (Bt) Cry, Cyt and Vip toxins are broadly used in pest control. Possible interactions (synergistic, additive or antagonistic) between these proteins can not only influence the overall efficacy of certain Bt-based bioinsecticide, but also raise questions regarding environmental safety. Here, we assemble, summarize and analyze the outcomes of experiments published over 30 years, investigating combinatorial effects among Bt Cry, Cyt and Vip toxins. We collected the results on 118 various two-to-five-component combinations that have been bioassayed against 38 invertebrate species. Synergism, additive effect and antagonism was indicated in 54%, 32% and 14% of experiments, respectively. Synergism was noted most frequently for Cry/Cyt combinations, followed by Cyt/Vip and Cry/Cry. In Cry/Vip combinations, antagonism is more frequent and higher in magnitude compared to other categories. Despite a significant number of tested Bt toxin combinations, most of them have been bioassayed only against one pest species. To aid the research on Bt pesticidal protein activity, we present TOXiTAXi (http://www.combio.pl/toxitaxi/), a universal database and a dedicated web tool to conveniently gather and analyze the existing and future bioassay results on biocidal activity of toxins against various taxonomic groups.

## Introduction

Biological plant protection products based on *Bacillus thuringiensis* (Bt) insecticidal proteins provide useful pest management tools to growers all over the world. Their implementation in agriculture, horticulture and forestry has not only granted efficient control of many economically important pest species, but also allowed to reduce the usage of harmful synthetic agents^1,2^. Bt-based bioinsecticides are currently the top selling biological plant protection products due to their high efficacy and safety to the environment and human health. Application of Bt biopesticides includes microbial formulations administered to target organisms by spraying, or Bt crops—genetically modified plants, producing Bt toxin proteins in their tissues^1–3^.

Bt toxins responsible for biocidal activity towards various invertebrate species include Cry, Cyt and Vip proteins^4^. Thus far, 271 Cry, 14 Cyt and 15 Vip holotype toxins were identified^5^ and named based on their level of amino acid sequence similarity. In this nomenclature^6^, every protein name (e.g., Vip3Aa59) starts with the Cry/Cyt/Vip abbreviation and the following four hierarchical ranks encoded by: number (primary rank), capital letter (secondary rank), lower case letter (tertiary rank) and two-digit number (quaternary rank). Accordingly, protein sequences with less than 45% amino acid similarity differ in primary rank (e.g., Cry1, Cry2, Cry3) and proteins with sequence similarity between 45–78% differ in secondary rank (e.g., Cry1A, Cry1B). The third rank (e.g., Cry1Ab, Cry1Ac) differentiates the protein sequences with 78–95% similarity, while the quaternary rank (e.g., Cry1Ac1, Cry1Ac3) groups protoxins sharing more than 95% similarity.

The differences in amino acid sequence between distinct Bt proteins have an impact on their level of toxicity and range of target species. Bt proteins are highly specific for their hosts—certain groups of toxins are only active against a limited set of taxonomic groups. For example, Cry1, Cry2 and Cry9 are active against lepidopterans, Cry3 against coleopterans, Cry4, Cry10 and Cry11 target dipterans, whereas Cry5 and Cry6 are toxic to nematodes^4^.

One of the major concerns regarding toxicity assessment of Bt toxins are the possible interactions (combinatorial effects) between them. It has been suggested that in some combinations the overall toxicity of Bt-toxin mixture may not be the sum of its constituents—they can also interact synergistically or antagonistically, thus significantly affecting the mortality of target insects. The concept and terminology of interactions among various agents (toxins) has been discussed for many decades and reviewed in a number of papers^7–9^. Accordingly, throughout this work we refer to the terminology and interaction estimation models/methods as follows. Additivity (additive effect) is the theoretical effect that is expected from the combination of multiple agents. This theoretical effect is estimated upon observed effects of combination constituents tested separately, usually using one of the two most common models: Loewe Additivity (simple similar action) and Bliss Independence (similar joint action). Any significant deviation from additivity is classified either as synergism (synergistic effect) or antagonism (antagonistic effect) based on the sign of difference (synergism has greater effect while antagonism has lower effect than expected additive effect). Examples of synergistic and antagonistic interactions have been described between different Cry toxins^10–13^, Cry and Cyt toxins^14,15^ and recently between Cry/Cyt and Vip toxins^16–21^. Given that the same toxin combination in different target species can act additively, synergistically or antagonistically—it is challenging to accurately predict the outcome without a direct bioassay. The prediction of toxin interactions is crucial i.e., in designing next generations of transgenic Bt-crops that produce more than one type of Bt toxin in their tissues. The proper selection of toxins in these so-called “pyramided Bt-crops” can have a serious impact on the level of control provided. Moreover, some concerns have been raised suggesting that synergistic interactions can pose a potential risk to non-target organisms^22,23^.

Due to possible application of Bt toxins in pest management, the number of reports on interactions between Cry/Cyt/Vip proteins is constantly growing, however the obtained records are highly non-uniform—each research group adopts different methodologies for toxin production and purification, bioassay length, toxicity parameters, interaction assessment, etc. It was reported that even small variation in bioassay can significantly influence the evaluation of toxin activity^24^. To date, the available results regarding activity of toxin compositions have not yet been assembled in a suitable database. Thus, the comparison and analysis of the toxin activity is highly inconvenient. In the past, a valuable database created by Kees Van Frankenhuyzen and Carl Nystrom was giving insecticidal activities of single Bt Cry and Cyt toxins (https://www.glfc.cfs.nrcan.gc.ca/bacillus). Despite its huge impact on the field^25^, the database did not cover toxicity reports on compositions and unfortunately it has not been available for many years now. Another perspective of the interaction evaluation studies is that recently, some concerns have been raised stating that combinatorial effects noted between Bt toxins in many works may be artifactual and synergism considerably overestimated^26^. Considering the above necessities, a summary and assessment of experiment results obtained so far is much needed.

Here we provide a comprehensive analysis of high-quality collection of all publicly available experiments concerning interactions between Cry/Cyt/Vip proteins. Throughout this work, any individual toxin-organism-result association is considered as a separate experiment. Additionally, for present and future applications we created a freely accessible database TOXiTAXi (http://www.combio.pl/toxitaxi/) to facilitate efficient management of growing experiment results concerning the activity, interaction types, and host range of Bt toxins. Tools created in this work will help to determine the potential of synergistic/antagonistic interactions in designing new biological pesticides. Moreover, TOXiTAXi was designed to be easily expandable and its flexibility enables processing of heterogeneous data regarding toxicity of various biocidal compounds with well-established or just potential use in pest control. The tools created in this work will be useful for both scientific environments and units involved in the design or practical use of plant protection products.

## Results

### Experiments concerning pesticidal toxins

We manually collected 1810 separate experiments that have been performed since 1993 and published in 76 research articles. Out of all collected experiments, 973 test biocidal activity of single toxins and 837 investigate the potency of toxin compositions. Among these, 1645 experiments (described in 59 manuscripts) investigate the activity of Bt proteins: 845 assess separate toxins and 800 concern Cry/Cyt/Vip toxin combinations. The remaining trials elucidate biocidal potency of agents other than Bt proteins including chitinases (e.g., enzymes characteristic for *Nicotiana tabacum* or *Serratia marcescens*), insect midgut proteins (e.g., *Spodoptera exigua* or *Manduca sexta* cadherins), botanicals (e.g., azadirachtin, pyrethroids), synthetic pesticides (e.g., neonicotinoids, carbamates), fungal spores (e.g., *Metarhizium anisopliae* or *Beauveria bassiana* spores) and scorpion toxins (e.g., AahIT, BjaIT). In total, the collected dataset includes experiments performed with: 131 various toxic agents, 54 invertebrate test species, 14 toxicity measures (e.g., LC_50_, LD_50_, IC_50_), 11 toxin preparation methods (e.g., purified proteins, parasporal crystals, inclusion bodies), and seven toxin administration methods (e.g., surface contamination, diet incorporation, leaf dip).

### Bt toxin combinatorial research analysis

**Figure 1:**
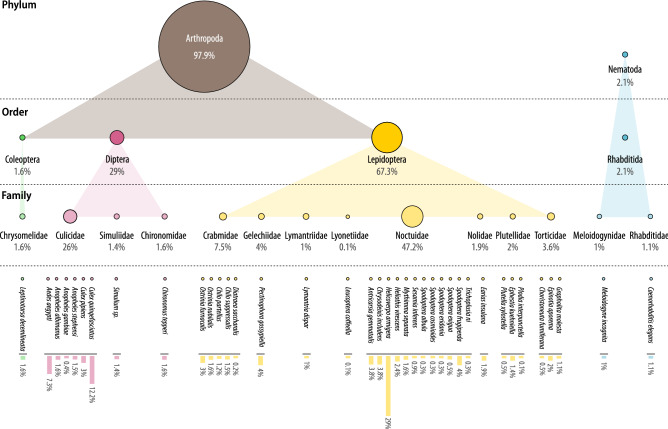
The share of individual taxonomic groups in experiments investigating interactions between Bt Cry/Vip/Cyt proteins. The size of the circles representing individual taxa corresponds to their percentage share in the total number of experiments. The data visualization has been performed using Inkscape v. 1.0 (https://inkscape.org).

The analysis of the collected data allowed us to assess the phenomenon of interactions between Bt Cry/Vip/Cyt proteins reported in research papers published for nearly 30 years. In Bt toxin combinatorial experiments, 50 different Bt proteins were used: Cry (82%), Vip (10%) and Cyt (8%). The insecticidal activity bioassays were performed using 38 test species representing two phyla (Arthropoda and Nematoda) and various classes, orders and families. The analysis shows that experiments are highly unequally distributed across taxa (Fig. 1). The highest number of arthropod species tested belong to Lepidoptera order (27 species), followed by Diptera (8 species) and Coleoptera (1 species). Within these orders, the most broadly represented is Noctuidae family (Lepidoptera), comprising 12 species. Invertebrates outside the Arthropod phylum, namely Nematodes, were only marginally tested (2 species). The number of experiments performed on each species is also highly uneven with lepidopterans being the most frequently tested (Fig. 1). The most overrepresented species is *Helicoverpa armigera* (present in almost every third experiment) followed by *Culex quinquefasciatus* and *Aedes aegypti*. Two most frequently tested families are Noctuidae and Culicidae, representing Lepidoptera and Diptera orders, respectively.

**Figure 2:**
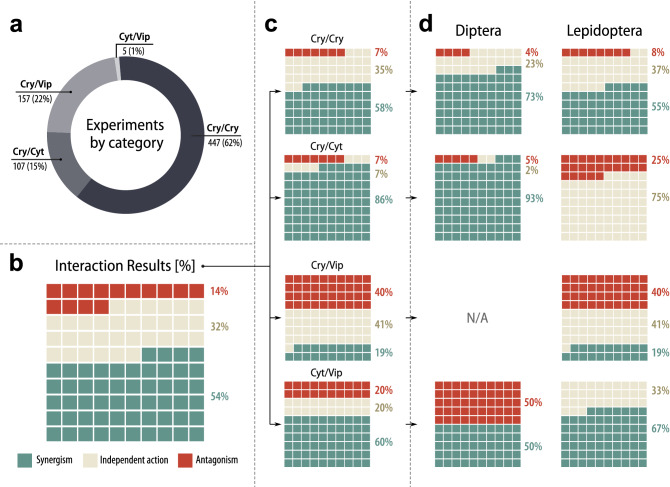
The experiments investigating interactions between Bt toxins: share of various composition categories in the total number of experiments (**a**); percentage of different interaction results totally (**b**) and in various composition categories (**c**); percentage of different interaction effects in various composition categories shown separately for bioassays performed on dipteran and lepidopteran species (**d**). *N/A* not applicable. The data visualization has been performed using Inkscape v. 1.0 (https://inkscape.org).

The dataset analysis shows that 118 different combinations between Bt toxins were created and tested for insecticidal activity. The vast majority are Cry/Cry combinations, followed by Cry/Vip, Cry/Cyt and Cyt/Vip (Fig. 2a). Out of 716 experiments investigating interactions between Bt proteins, the greater part (54%) indicate synergism, then additive effect (32%) and the rest (14%) assume antagonism between components (Fig. 2b). Therefore, the ratio of synergistic interactions to sum of additive and less than additive interactions is almost equal. However, the share of each interaction type depends strongly on the toxin combination category analyzed (Fig. 2c). For example, the rate of synergism in Cry/Cry, Cry/Cyt, Cry/Vip and Cyt/Vip compositions is 58%, 86%, 19% and 60%, respectively. Therefore, it appears that in Cry/Cyt combinations synergism is generally much more evident compared to the remaining categories. In Cry/Vip combinations however, synergistic interactions are a minority and the magnitude of SF is relatively low, whereas antagonism is present in nearly 40% of recorded data and the SF values calculated for antagonistic interactions are lower than in other mixture categories. Note however, that the dataset includes additional 84 experiments reported using Bt toxin mixtures, but no interaction type was determined by their authors.

Also, substantial differences exist between combinatorial effects estimated in bioassays performed on dipteran and lepidopteran species (Coleoptera and Rhabditida orders were omitted due to small number of experimental data). It is evident that toxins assayed against dipteran species much more often act synergistically compared to toxins assayed against lepidopterans (Fig. 2d).

**Figure 3:**
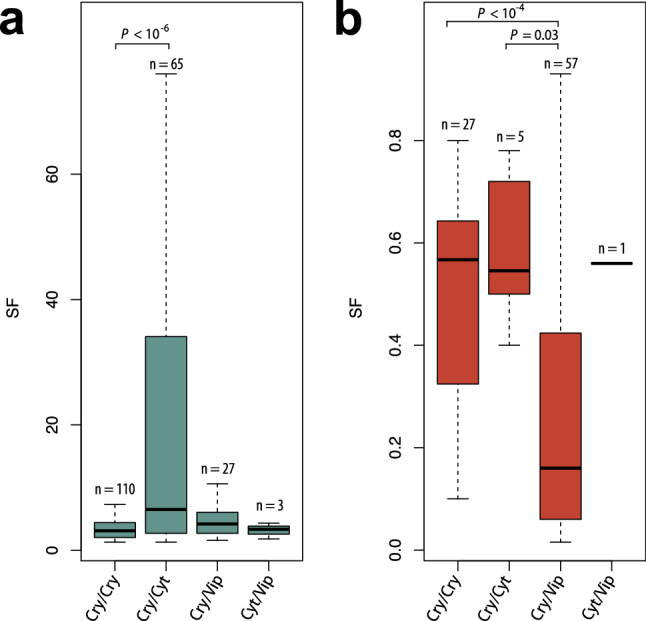
The magnitude of synergism (**a**) and antagonism (**b**) in reported experiments investigating combinatorial effects between Bt toxins, shown separately for different composition categories. Box indicates range from first quartile (25%) to third quartile (75%) and the median (horizontal line within each box) of reported SF values; whiskers indicate lowest and highest values. SF value of 455 for synergistic interaction in one Cry and Cyt mixture has not been included for the sake of clarity. Sample sizes (number of experiments with SF specified) are indicated for combinations at the top of each bar. Connecting lines indicate differences between populations (two-sided Mann–Whitney U-test, *P* < 0.05). The data visualization has been performed using Inkscape v. 1.0 (https://inkscape.org).

Out of 716 experiments elucidating combinatorial effects between Bt toxins, 402 estimate the synergism factor (SF) parameter. The SF values vary greatly depending on tested toxin composition. For example, in the experiments that reported synergism, SF lies between 1.3 and 455, however in the majority of experiments the SF values are in the range between 2.35 (first quartile) and 6.41 (third quartile) with median at 3.8. In the experiments indicating antagonism SF ranges between 0.02 and 0.93, while most of the results point SF around 0.11 (first quartile) and 0.58 (third quartile) with median at 0.32. Analysis of SF values in different composition categories (i.e., Cry/Cry, Cry/Cyt, Cry/Vip, Cyt/Vip) shows highly uneven distribution (Fig. 3a,b). Synergism estimated for Cry/Cyt combinations is higher in magnitude compared to other compositions. By contrast, antagonism estimated for Cry/Vip combinations is the lowest of all mixture categories.

**Figure 4:**
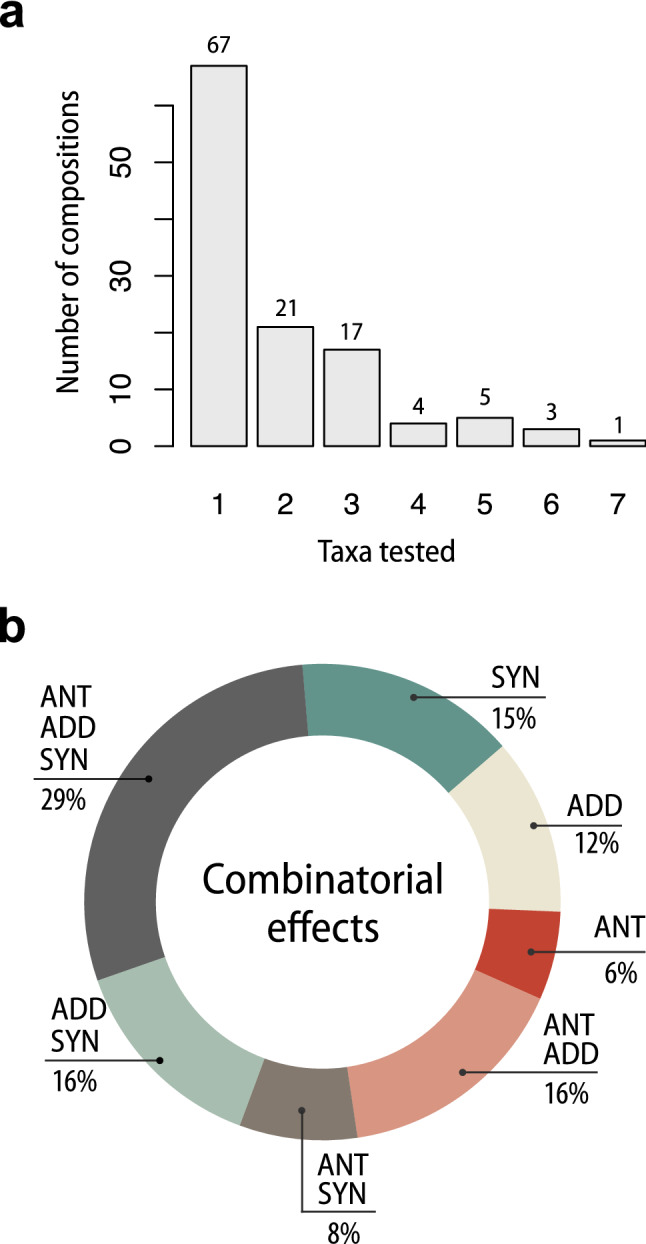
The relationship between the number of tested Bt-toxin combinations and the number of invertebrate species against which they have been bioassayed to date (**a**) and variability of interactions noted for combinations tested against more than one species (**b**). *SYN* synergism, *ADD* additive effect, *ANT* antagonism. The data visualization has been performed using Inkscape v. 1.0 (https://inkscape.org).

The activity of toxin combinations across all experiments was assessed against different number of test species (Fig. 4a). The most frequently tested is Cry1Ab + Cry1Ac composition, assessed against 7 organisms, but the vast majority of the compositions were tested against only one insect species. Moreover, high variability of combinatorial effects is noted for mixtures tested against two and more taxa (Fig. 4b). Only for 33% of these combinations the same combinatorial effect is noted, whereas for the majority of mixtures the interaction type was different depending on the tested organism. The complete list of Bt toxin combinations along with the corresponding tested species is available in Supplementary Dataset S1.

In general, two-component mixtures are the most frequently assessed (92.1% of experiments), however, compositions containing 3, 4, and 5 components are also present in 6.9%, 0.4% and 0.6% of experiments, respectively. Notably, the number of mixtures containing more than two constituents is growing over time (data not shown).

To determine the interaction type between Bt toxins, the available reports used three approaches. The most frequent is a simple similar action model (55.8% of trials), and independent joint action model (36.1%), i.e., used in works performed by Tabashnik^27^ and Fernandez-Luna et al.^15^, respectively. Third approach is somewhat intuitive and generally relies on comparison of effects exerted by composition and individual constituents, where one constituent is often inactive or sublethal doses are administered to tested organism—these various empirical methods were applied in a minority of experiments (7.3%), i.e., described by Kaikaew et al.^28^.

To assess the prevalence of comparative data we have determined the number of independent research teams investigating interactions between given toxins, using the same species as the test organism. The experiments were considered to have been performed by independent teams when they were published in journal articles, in which none of the authors was mentioned twice. This survey showed that only ten toxin combinations out of 118 were tested against certain target species by more than one team (Table 1). In addition, nine out of ten combinations were tested only by two independent teams and one mixture was tested by three research groups. Experiments elucidating the interactions between toxins characteristic for *B. thuringiensis* subsp. *israelensis* mostly agreed on synergism (e.g., Cry4Aa + Cry4Ba; Cry4Aa + Cry4Ba + Cry11Aa; Cyt1A + Cry11A; Cry4Ba + Cyt1Aa; Cyt2Aa2 + Cry4Ba; Cry11Aa + Cyt1Aa), with the exception of Cry4Ba + Cry11Aa, where contradictory results were obtained by two teams. On the other hand, the results of experiments performed on lepidopteran-active toxin mixtures (Cry1Ac + Cry1F, Cry1C + Cry1Aa, Cry1Ac + Cry2Ab) were always divergent.

**Table 1:** Bioassays investigating the same toxin combination and target species performed by more than one research team. N/A—not applicable.

Bt toxin combination	Target species	Estimated combinatorial effect [source article]		
		Team A	Team B	Team C
Cry4Aa + Cry4Ba	*Aedes aegypti*	Synergism^55^	Synergism^56, 57^	N/A
Cry4Aa + Cry4Ba + Cry11Aa	*Aedes aegypti*	Synergism^58^	Synergism^57^	N/A
Cyt1A + Cry11A	*Aedes aegypti*	Synergism^59^	Synergism^58^	Synergism^14, 60^
Cry4Ba + Cry11Aa	*Aedes aegypti*	Synergism^58^	Antagonism/additivity^57^	N/A
Cry4Ba + Cyt1Aa	*Aedes aegypti*	Synergism^58^	Synergism^61^	N/A
Cyt2Aa2 + Cry4Ba	*Aedes aegypti*	Synergism^62^	Synergism ^28^	N/A
Cry11Aa + Cyt1Aa	*Culex quinquefasciatus*	Synergism^63^	Synergism^64–66^	N/A
Cry1Ac + Cry1F	*Helicoverpa armigera*	Synergism^67^	Additivity^12^	N/A
Cry1C + Cry1Aa	*Helicoverpa armigera*	Synergism^11^	Additivity^13^	N/A
Cry1Ac + Cry2Ab	*Helicoverpa armigera*	Synergism/additivity^12^	Antagonism/additivity/synergism^68^	N/A

### A web resource for toxicity of *Bacillus thuringiensis* protein compositions

To supplement the results of this study, we have launched a novel, publicly accessible web application, TOXiTAXI (http://www.combio.pl/toxitaxi/) that conveniently gathers and analyzes the existing and future bioassay results on biocidal activity of toxins against various taxonomic groups. The TOXiTAXi database interface is designed to be used by a bench scientist on an everyday basis. Following a simplicity rule, the interface of TOXiTAXi has been built on only one result window and simple two types of querying systems, despite the vast amount and variability of data contained in the database.

The data selection process is provided in the form of a hierarchical expandable menu to supply the functionality for selecting any combination of toxins or target species. In this way, users can select toxins either by marking individual proteins (e.g., Cry1Ab3 and/or Vip3Aa20) or groups of proteins (e.g., all Cry and/or Vip proteins) from the tree-like menu reflecting the proteins hierarchical nomenclature or by typing protein names in the input text box. Analogously, users can select individual target species of interest (e.g., *Helicoverpa armigera*) or different combinations of taxonomic groups (e.g., all Lepidoptera and Coleoptera species). The real potential of the TOXiTAXi service is based on its ability to combine different datasets into one display. There is no limit as to the number and type of selected toxins and target species that can be combined in a single query.

**Figure 5:**
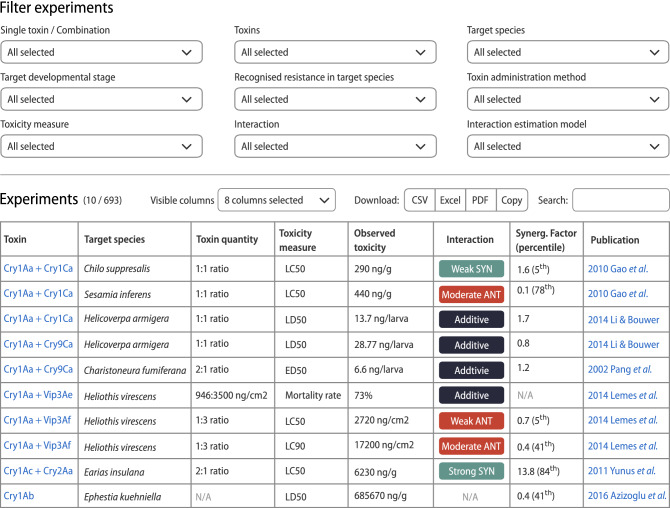
Example of TOXiTAXi result window for bioassays including various *B. thuringiensis* Cry/Vip toxins. The figure has been created using Inkscape v. 1.0 (https://inkscape.org).

The experiments found for a given toxin/combination of toxins or target species are summarized in the table (Fig. 5) providing basic information such as: toxin quantity, observed toxicity including biocidal activity measure (e.g., LC_50_, LC_90_, and mortality rate) and its unit (e.g., ng/cm^2^), combinatorial effect among toxins in composition (i.e., additivity, synergism, and antagonism), and reference publication. The table is searchable and sortable, allowing users to quickly filter the experiments with a desired span of experiment parameters, for example experiments focusing on *Spodoptera frugiperda* as a target species, bioassay type of LC_50_ and synergistic interaction. Users can also generate customizable and integrated results by adding additional information concerning experiments such as: (i) the developmental stage of target species (e.g., larval instar); (ii) the recognized resistance in target species (e.g., the resistance to Cry1Ac protein); (iii) bioassay duration (e.g., 7 days), (iv) toxin administration method (e.g., surface contamination); (v) the expected toxicity (theoretical toxicity value of toxin combination assuming lack of synergism and antagonism); (vi) confidence intervals of the observed toxicity; (vii) the synergism factor (SF; the ratio of expected to observed toxicity) and (viii) estimation model used to determine the interaction among toxins. Such customized tables can be further downloaded from the web page in common tabular formats (i.e. Excel, CSV, and PDF files).

The detailed results for a single experiment are presented in a record window in a form that is divided into three distinct sections. The first section is dedicated to information regarding the individual components of a given toxin or combination of toxins (e.g., Cry1Ac1 + Cry9Aa). When applicable, the section also provides details on toxin modification, preparation and expression host. The second and third record sections provide information on target species and experiment results, respectively.

### The magnitude of synergism and antagonism

One of the main purposes of the TOXiTAXi web application is to provide the user with a tool for comprehensive analysis of combinatorial effects among toxins. The theoretical basis and model assumptions for toxin interactions (not limited to insecticidal toxins) are subject of academic debates since the early twentieth century^7–9,27,28^. One of the discussed aspects is the SF value threshold above which synergism may be assumed. For example, some recent works^26,29,30^ suggest that SF value should be at least 2 to “consider a result as being more than additive”. The authors also state that more than tenfold increase in activity due to synergism is observed in bioassays very seldom. To acknowledge this important issue and to improve information display for the user, a categorization of the magnitude of combinatorial effects (both synergistic and antagonistic) has been implemented. For this purpose, a SF scale was used, where the “strength”of synergistic interactions is classified into three categories: “weak/doubtful synergism” (SF ∈ [1, 2)), “moderate synergism” (SF ∈ [2, 10)), and “strong synergism” (SF ∈ [10, ∞)), shown on the TOXiTAXi website in the “Interaction” column of the result window. Analogously, we used a SF scale, where the magnitude of antagonistic interactions is classified into three categories: “weak/doubtful antagonism” (SF ∈ [0.5, 1)), “moderate antagonism” (SF ∈ [0.1, 0.5)), and “strong antagonism” (SF ∈ [0, 0.1)), depicted on the TOXiTAXi website in the “Interaction” column of the result window. Since the proposed thresholds are fixed and thus can only provide a rough estimate of interaction strength, we also implemented a manifestation of SF values in the form of percentiles, which are dynamically generated on the website based on the distribution of all SF values currently deposited in the database, separately for synergistic and antagonistic effects. Percentiles as a measure of interaction strength inform the user about a relative standing of a given SF in comparison to all synergistic or antagonistic interactions present in the database. For example, the synergism factor reported for Cry1Ac + Cry1F (SF = 26.3) scores above the 90th percentile, which places the Cry1Ac + Cry1F interaction among the top 10% of synergistic interactions stored in the database. The percentiles are shown in the “Synergism factor” column of the result tree, following the SF values.

## Discussion

Entomopathogenic properties of *B. thuringiensis* were observed as early as in 1901, however first genes encoding Bt insecticidal toxins were cloned and sequenced more than 80 years later^2^. Because of the fast development of genetic engineering, to date we know the sequences of more than 350 Cry, Cyt and Vip toxins—main molecules responsible for Bt virulence towards target organisms. Some of them were heterologously expressed and separately assessed for their biological activity, but the toxins were usually tested on a rather limited number of taxa^25^. Despite a great number of toxicity data obtained so far, still much is to be revealed in this field. Recently however, the attention has been focused on investigating the pesticidal potency of compositions containing various sets of Cry/Cyt/Vip proteins. These extensive studies were employed especially after the development of new generations of pyramided Bt-crops—genetically modified plants expressing more than one Bt toxin type in their tissues. Multiple toxins used simultaneously as bioinsecticides have a great advantage over single-protein agents because they can control a broader range of target species^29^ and prevent/delay insect resistance development^30^. It was proposed that between different Bt toxins, synergistic or antagonistic interactions can occur—toxicity of a toxin set may not be a simple resultant of constituent potency. This phenomenon could have a tremendous impact on designing and developing multi-toxin bioinsecticides. Properly selected Bt genes can be “stacked” in the transgenic event to achieve better efficacy against target pests, resulting from synergism. On the contrary, combined toxins with antagonistic interactions could significantly lower the level of control. Another critical aspect of toxin assemblies is the possibility that synergism between toxins can influence non-target species, despite the constituents tested separately did not pose any threat. Unfortunately, investigations performed prior to Bt-crop registration did not include interaction estimation between Bt-toxins^23^. Currently only 50 out of 300 known holotype Cyt/Cry/Vip proteins were investigated for their mutual interactions. It can be anticipated that in the future, much more related information will be gathered. Therefore, the effortless assembly and interpretation of toxicity bioassay data is of great importance.

The information on biological activity of Bt proteins is very difficult to analyze in a comprehensive manner, because the extensive data is greatly scattered across the literature. In this study we analyzed 59 manuscripts investigating potency of Cry/Cyt/Vip compositions. A considerable diversity of experimental approaches exists in the available works, therefore, the bioassay results are confounded by various factors such as: toxin types tested; protein expression, purification and modification protocols; bioassay duration; toxin administration to test organism, etc. To overcome these issues and allow convenient data analysis, a suitable database is necessary. In this work, dedicated tools were created to easily assemble, summarize and analyze the data on biological potency of toxins. Results of experiments on insecticidal activity of Bt Cry/Cyt/Vip protein mixtures were gathered and incorporated into the database, along with analogous data for individual toxins, present in the studied articles. The accompanying internet application enables easy access to assembled data and selection of desired information.

We have used the newly created tools to analyze the current state of knowledge regarding interactions between Bt protein toxins. The analysis shows that experiments are highly unequally distributed across taxa. A strong overrepresentation of studies investigating Bt toxin composition activity involving lepidopterans can be noted, in comparison to results on the activity of single Cry and Cyt toxins^25^. The probable cause is the prevalence of existing and newly emerging pyramided genetic events designed to provide resistance to lepidopteran insects of genera such as *Helicoverpa*, *Spodoptera*, *Ostrinia*, etc^31,32^. Therefore, the laboratory tests involve the same species to assess the toxin performance in the controlled environment. The natural consequence of chosen target species is also the uneven distribution of Bt toxins investigated. The majority of tested proteins belong to lepidopteran-active Cry1 and Cry2 families, and in recent years much higher percentage of compositions including Vip3 proteins were tested. This trend likely reflects the emergence and increase of the market share of genetic events expressing Vip3 toxins such as: Bollgard 3, TwinLink Plus, WideStrike 3 cotton varieties or Agrisure Viptera 3220 corn^31,33^. It can be anticipated that along with the growing number of pyramided Bt-crops also the number of reported results on biocidal activity of various Bt-toxin compositions will follow rapidly.

Recently, some concerns have been raised that many available research results regarding Bt toxin combinatorial effects may be affected by different types of errors (see below), especially when noting a synergistic effect^26^. To support this conclusion, the authors argument that: “i) experiments reporting synergistic interactions are a minority, ii) the degree of synergism reported is low in magnitude, iii) reported interactions are sometimes equivocal/inconclusive due to unconfirmed model assumptions or other bioassay challenges, and iv) due to biological response variation many of the reported interactions may be artefactual”. The above-mentioned article focuses on lepidopteran-active Cry1, Cry2, Cry9 and Vip3 protein combinations. When analyzing part of TOXiTAXi content with roughly equivalent data selection criteria (namely the experiments investigating Cry/Cry and Cry/Vip combinatorial effects between Bt toxins tested on lepidopteran larvae), the results show 43.6%:66.4% ratio of synergistic to additive plus less than additive interactions (data not shown). This is almost identical to results obtained by Walters et al.^26^. Moreover, generally a low magnitude of noted SF values has been shown in both works. However, when analysis is performed on larger dataset and especially on different combination categories, a very complex and diverse picture appears. It is evident that in Cry/Cyt combinations synergism is generally much more evident compared to the other categories—judging on number of experiments noting synergistic interaction as well as on synergism magnitude. In the experiments involving Cry/Cry and Cyt/Vip combinations, synergistic interactions are a majority, however the SF values are relatively low in magnitude, although the latter category is represented by a small number of experiments. In contrast, a high percentage of antagonistic effects in Cry/Vip compositions is evident and the calculated SF values in assays noting antagonism between Cry and Vip toxins are lower than in other composition categories. Therefore, our comparison indicates that size and selection criteria of the dataset can have significant impact on analysis of interactions between Bt-toxins. Moreover, differences between various categories of compositions as well as tested taxa should be taken into consideration in future studies. It was not a goal of this work to address other matters related to the correctness of approaches utilized to estimate the combinatorial effects between Bt toxins, such as fidelity of interaction assessment models or biological response variation. We would like to look at the Bt-toxin interaction data from a different perspective and highlight another possible challenge. After analysis of our database, it can be observed that in most cases a particular toxin combination was tested against one insect species only by one research team (and furthermore, usually reported in one article). There are only ten exceptions from this general trend, but even in these instances a certain combination-species association is usually tested only by two independent teams. Among them, experiments investigating interactions between dipteran-active toxins derived from *B. thuringiensis* subsp. *israelensis* are quite consistent and indicate synergism in most cases. On the other hand, experiments performed with lepidopteran-active toxins on *Helicoverpa armigera* state different interactions in each investigating team. Experience gained from insecticidal activity testing for single toxins suggests that experimental conditions varying between labs can give divergent results. One example of this fact is the insecticidal activity of Cry2Ab toxins towards *Spodoptera exigua*. In some works the proteins were found effective^34,35^, but other teams show their marginal potency^36^ or no activity at all^37^ against tested lines of *S. exigua*. Therefore, the available comparative data on Bt toxin combinatorial effects is quite limited, and may not be sufficient to draw reliable conclusions. Moreover, the mechanisms underlying the synergistic/antagonistic interactions between Bt toxins are still only hypothetical^23^, which further adds uncertainty to the area. Concluding, more research is needed to gain confidence regarding the occurrence of interactions between different Bt toxins, and to properly assess the scale of this phenomenon. By successive incorporation of the results generated in the future, the database created in this work should help to monitor this issue.

As described above, the dataset assembled in this work generally included research papers investigating insecticidal potency of combinations containing Bt Cry/Cyt/Vip proteins and interactions between constituents. However, single toxin activity results available in the manuscripts were also incorporated into the database. Currently, TOXiTAXi is the only available bioinformatic tool gathering this type of information. In the past one such database existed, organizing research experiments investigating the biological specificity of individual Bt delta-endotoxins Cry and Cyt^25^ but unfortunately it is no longer available, and the toxicities of protein combinations were not included in its dataset. Currently, the database created in this work contains only a limited portion of single Bt toxin specificity info available in the literature, however it is perfectly suitable to process these types of result. In the future, it can be expanded to include a full set of data. Additionally, in order to test the universal potential of the database, the results regarding biocidal activity of agents other than Bt Cry/Cyt/Vip proteins was incorporated into the dataset. The information includes pesticidal activity of enzymes; insect midgut proteins; biochemical substances and synthetic pesticides. The input data could be conveniently incorporated into the database without any difficulties and loss of information, whereas the web application can provide suitable display and analysis of this information in the same manner as for Cry/Cyt/Vip proteins. In addition, the hierarchy in the expandable tree available for the user can be easily rearranged when new data will demand changes in the ranks of insecticide types. Therefore, the use of the tools created in this work is not limited to Bt protein toxins, but enables incorporation of information on highly heterogeneous substances with biocidal activity.

In conclusion, the created database is the first comprehensive assembly of existing experimental data on Bt toxin combinatorial effects. This database along with the dedicated web tool can be used to further accumulate the existing, as well as future results on toxicity of *B. thuringiensis* toxins and other biocidal agents of various origins. The application enables effortless analysis of precisely selected, desired information. Tools created in this work can be used not only by the academic community but also units involved in implementation of new solutions and products in the field of biological plant protection. The gathered dataset will be updated soon after new pesticidal toxin combinatorial effects are reported in the literature. To better streamline the process of data acquisition and enhance usability of the TOXiTAXi database, we encourage users to submit their feedback and suggestions of new studies to include, so that we can continue to improve this web resource, tailoring it to the specific needs of the biocontrol-focused community.

## Materials and methods

### Experiment source selection

Research papers investigating interactions between Cry, Cyt and Vip toxins were selected with Scopus database. The search was performed using different combinations of keywords: Cry; Cyt; Vip; thuringiensis; interaction; synergistic; synergism; antagonistic; antagonism. From the initial dataset further exploration was made selecting the manuscripts, which were written in English and contained experiments: (i) investigating the insecticidal activity of Cry/Cyt/Vip toxin compositions; (ii) performed using *B. thuringiensis* Cry, Cyt or Vip toxins obtained through expression of cloned genes in hosts not producing other pesticidal toxins (typically *E. coli* or acrystalliferous *B. thuringiensis* strains); (iii) providing proper quantification of used toxins. Additionally, some papers not found in Scopus database but appearing in the bibliography of selected articles have been included in the data set, if they met the above-mentioned criteria. In total, 58 manuscripts were initially included in the dataset. From a selected publication, the available results of experiments investigating toxicity towards invertebrates were incorporated into the database. This includes activity of toxin compositions as well as separate Cry/Cyt/Vip toxins. Each recorded experiment includes information regarding: (i) source publication (authorship, publication date, PubMed ID); (ii) tested toxic agent/composition (exact toxin name(s) according to current nomenclature, toxin source, expression host, molecular modifications applied, details on toxin preparation); (iii) tested target organism (species name; strain/laboratory line, developmental stage, recognized resistance to toxins); (iv) bioassay (toxin administration method, bioassay duration, toxicity measure, observed toxicity with units and confidence intervals); and (v) interaction among toxins (expected toxicity of a mixture, interaction type, estimation model of interaction, and synergism factor).

The published bioassay data were incorporated into the database without alterations with the following exceptions: (i) if synergism factor was not provided by the authors, it was calculated when possible; (ii) antagonism factors were recalculated to synergism factors; (iii) toxicity measure units were recalculated to nanograms (e.g., µg/cm^2^ to ng/cm^2^, µg/ml to ng/ml, etc.). Experimental records were omitted when toxicity bioassays were performed using: wild type Bt strains (usually containing sets of not precisely characterized and/or non-quantified toxins); binary toxins such as Tpp (formerly Bin), Gpp34/Tpp35 (formerly Cry34/Cry35), Vpb1/Vpb2 (formerly Vip1/Vip2); *Lysinibacillus sphaericus* toxins (e.g. Mtx, Mpp2-4); Bt toxins obtained from transgenic crops—regardless if the above were tested with or without accepted Cry/Cyt/Vip toxins. Moreover, if for some reason the exact values could not be determined for bioassay parameters (e.g. results presented solely in a chart form), the data was not included.

In order to test the broad-use potential of the created database, a selection of arbitrarily chosen manuscripts were additionally added to the dataset. These works investigate biocidal activity of agents other than *B. thuringiensis* Cry/Cyt/Vip proteins, including: chitinolytic enzymes^38–42^, synthetic and biochemical insecticides^43–45^, insect midgut proteins^46–48^ *Bacillus thuringiensis* Mpp5A (formerly Sip1 proteins)^49,50^, scorpion toxins^51^ and fungal spores^52,53^.

### Toxin nomenclature

All publications incorporated into the TOXiTAXi dataset use nomenclature developed in 1998^6^ for naming *B. thuringiensis* insecticidal proteins. Recently however, a revised nomenclature has been proposed^54^. Toxins included into the TOXiTAXi database have identical names in both nomenclatures, with the exception of Cry6-type and Cry55-type proteins, currently named App6-type and Xpp55-type, respectively^5^. For the sake of clarity, in the analyses presented in this study, these few proteins are considered as Cry. In the web application however, both names (old and new) are given, when applicable.

In the current nomenclature, the quaternary rank groups identical or almost identical (a few amino acid substitutions) Bt proteins and the differences are considered biologically insignificant^6,25^. Therefore, throughout this work, toxins are considered unalike, when they differ in tertiary rank.

### IT technology

The backend of TOXiTAXi is implemented in Python with Django 2.2 web framework (https://www.djangoproject.com), dynamically generating web pages using Apache2 with WSGI. The data are stored using a MySQL database, and a modular database schema allows further growth and incorporation of new data types. The web pages are constructed using HTML5, CSS3, JavaScript with jQuery library (https://jquery.com) and Bootstrap 4.4 framework (https://getbootstrap.com). Dynamic and interactive elements of the taxon page are developed using SVG markup language with the d3.js library (https://d3js.org) for toxin visualization, and DataTables (https://datatables.net) as a tabular feature viewer, respectively.

### Ethical approval

This article does not contain any studies with animals performed by any of the authors.

### Informed consent

Informed consent was obtained from all co-authors included in the study.

## Supplementary information

Supplementary Information.
